# Preparing for implantation

**DOI:** 10.7554/eLife.73739

**Published:** 2021-10-18

**Authors:** J Julie Kim

**Affiliations:** Division of Reproductive Science in Medicine and Center for Reproductive Science, Department of Obstetrics and Gynecology, Northwestern University Chicago United States

**Keywords:** endometrium, embryo implantation, organoid, assembloid, senescence, decidualisation, Human

## Abstract

A new laboratory model helps to understand the role of senescent cells in fostering a uterine environment that can support an embryo.

**Related research article** Rawlings TM, Makwana K, Taylor DM, Molè MA, Fishwick KJ, Tryfonos M, Odendaal J, Hawkes A, Zernicka-Goetz M, Hartshorne GM, Brosens JJ, Lucas ES. 2021. Modelling the impact of decidual senescence on embryo implantation in human endometrial assembloids. *eLife* **10**:e69603. doi: 10.7554/eLife.69603

The endometrium, the structure that lines the womb, is one of the most fascinating tissues in the human body. Every month, it grows, changes and destroys itself under the influence of the ovarian hormones estrogen and progesterone. In particular, it undergoes a series of modifications, known as decidualization, which include new types of endometrial cells emerging to help the embryo implant and survive until the placenta takes over. This ‘receptive’ phase only lasts a few days and takes place after ovulation, when progesterone levels are high.

How the endometrium can select and support an embryo has been widely researched, especially in the context of assisted reproductive technologies, recurrent miscarriages and implantation failure. Yet, given the ethical considerations of reproductive experiments and the lack of appropriate model systems, scientists still do not have a full grasp on how endometrial decidualization enables implantation.

For decades, the biology of the endometrium has primarily been studied in the laboratory using cells taken from the uterus after surgery. However, due to the way different cell types grow in dishes, certain endometrial cells are better understood than others. For instance, more is known about the stromal cells (which make up the supporting ‘connective’ tissue) than about the epithelial cells which line the endometrium and form glands secreting essential factors.

The recent emergence of organoid cultures that can mimic native tissues in the laboratory may provide a new source of information. Indeed, certain research groups have established ways to grow epithelial cells in three dimensions, while others have created organoids that comprise both epithelial and stromal cells (Wiwatpanit et al., 2020; Cheung et al., 2021; Boretto et al., 2017; Turco et al., 2017). Now, in eLife, Jan Brosens and colleagues – including Thomas Rawlings as first author – report a new laboratory model of the endometrium that can be used to explore the endometrial changes required for embryos to implant (Rawlings et al., 2021).

The team (which is based at the California Institute of Technology, University Hospitals Coventry and Warwickshire NHS Trust, and the Universities of Cambridge and Warwick) generated ‘endometrial assembloids’ that contained both stromal and epithelial cells growing in three dimensions. The cells were able to organize themselves in a manner that resembles the architecture of an actual endometrium, with gland-like structures surrounded by a bed of stromal cells.

A cocktail of ovarian hormones was applied to the assembloids for four days in order to induce decidualization. Single-cell RNA sequencing was then performed to identify different cell populations that had emerged as a response. Surprisingly, after hormone treatment, there were multiple subpopulations of stromal and epithelial cells. All cell populations exhibited unique patterns of gene expression that mapped to a specific phase in the menstrual cycle, including the receptive phase during which the endometrium can welcome an embryo.

Assembloids that had not been exposed to the hormones featured three subtypes of epithelial cells, and two types of stromal cells. However, the assembloids that had undergone decidualization carried three types of epithelial cells and three types of stromal cells: this included, as Rawlings et al. had predicted, a population of epithelial and stromal cells which had become senescent under the influence of the hormone cocktail. In this ‘suspended’ state (which is often associated with aging), cells are unable to divide but they remain biologically active and can release harmful factors that damage neighboring cells. However, senescence in the endometrium may actually be necessary for implantation to take place, a question that Rawlings et al. could explore with their new model.

The team first used a publicly available computational tool to analyze single-cell RNA data: their goal was to assess how the different cell subpopulations interact, and to predict interactions between surface receptors and their ligands. This showed intense communication between epithelial and stromal cell types; more interestingly, subsets of decidual stromal cells communicated with one another resulting in the activation of the tyrosine kinase signaling pathway, which controls many cellular processes. Exposing the assembloids to dasatinib, a cancer drug which inhibits this pathway, eliminated the emergence of decidual stromal cells that were senescent, while increasing the number of non-senescent decidual cells.

This approach allowed Rawlings et al. to model and influence senescence in order to assess its impact on early embryo development. Human embryos cultured in the presence of assembloids not exposed to dasatinib (and therefore containing senescent decidual stromal cells) could increase in diameter and move. However, assembloids treated with dasatinib appeared to restrict embryo growth and movement (Figure 1).

**Figure 1.**
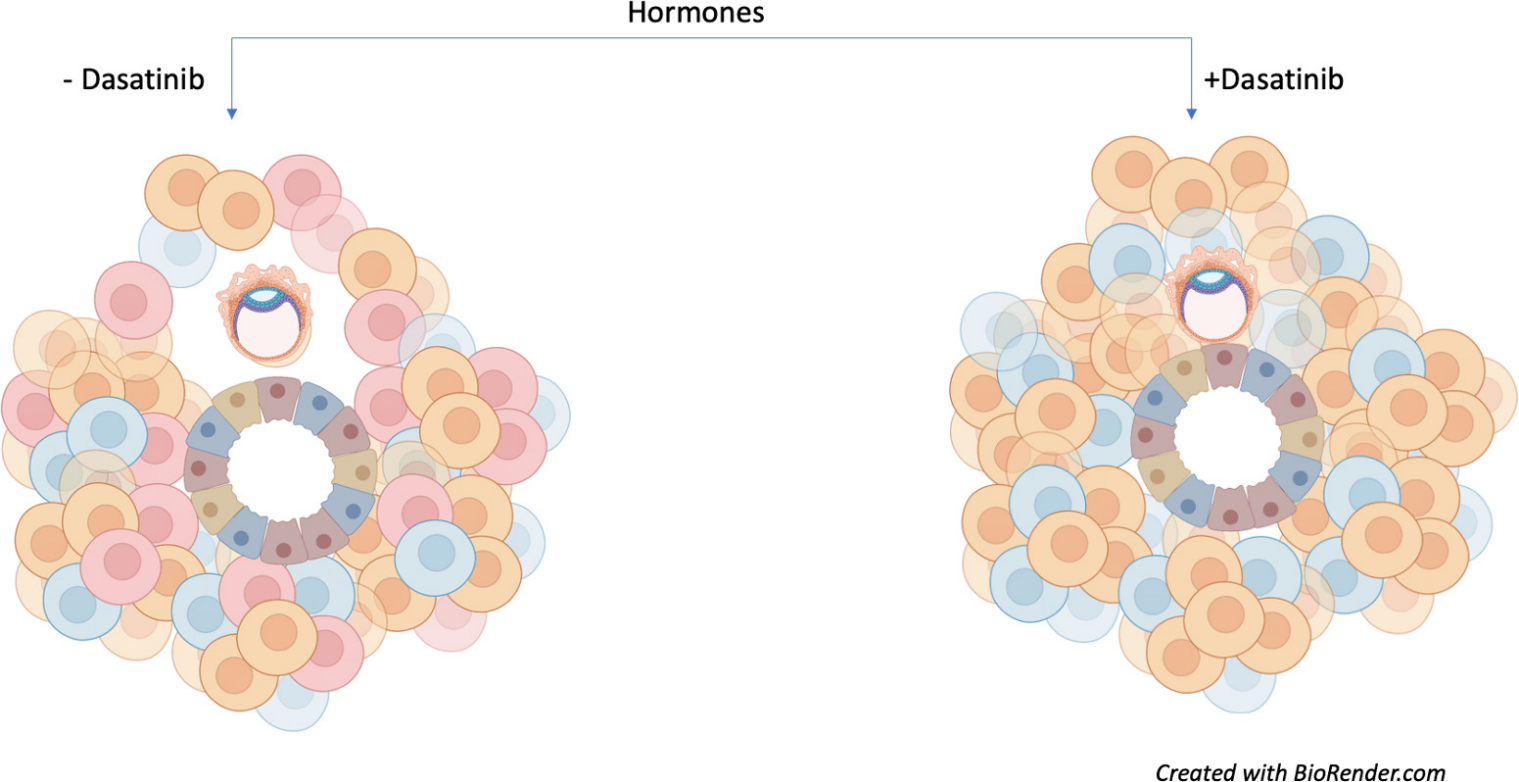
Senescent decidual cells promote embryo expansion and movement. Rawlings et al. developed endometrial assembloids and exposed them to hormones to help their differentiation process. These models contain both stromal (round) and epithelial (trapezoid) cells. The three subpopulations of stromal cells are shown (predecidual in blue; decidual in orange; and senescent decidual in red). Assembloids not treated with the tyrosine kinase inhibitor dasatinib (left) promote the expansion and movement of an embryo (oval-shaped structure). Treatment with the drug (right) causes senescent decidual cells to be eliminated from the assembloid, which prevents the expansion of the embryo and restricts its movement.

Image credit: Image created with Biorender.com.

The work by Rawlings et al. highlights the importance of establishing models that closely mimic human physiology, even if it means co-culturing multiple cell types together. In turn, state-of-the-art technologies such as single-cell RNA sequencing – and their associated bioinformatic tools – can deconvolute this complexity one cell at a time. As implantation remains a poorly understood event, these new approaches could finally help to unravel the complex mechanisms that shape the endometrium for pregnancy.
